# Identification of an *N*-acetylneuraminic acid-presenting bacteria isolated from a human microbiome

**DOI:** 10.1038/s41598-021-83875-w

**Published:** 2021-02-26

**Authors:** Zhen Han, Peter S. Thuy-Boun, Wayne Pfeiffer, Vincent F. Vartabedian, Ali Torkamani, John R. Teijaro, Dennis W. Wolan

**Affiliations:** 1 Department of Molecular Medicine, The Scripps Research Institute, La Jolla, CA 92037 USA; 2 San Diego Supercomputer Center, University of California, San Diego, La Jolla, CA 92093 USA; 3 Department of Immunology and Microbiology, The Scripps Research Institute, La Jolla, CA 92037 USA; 4 Scripps Research Translational Institute, The Scripps Research Institute, La Jolla, CA 92037 USA; 5 Department of Integrative Structural and Computational Biology, The Scripps Research Institute, La Jolla, CA 92037 USA

**Keywords:** Microbial communities, Glycobiology

## Abstract

*N*-Acetylneuraminic acid is the most abundant sialic acid (SA) in humans and is expressed as the terminal sugar on intestinal mucus glycans. Several pathogenic bacteria harvest and display host SA on their own surfaces to evade Siglec-mediated host immunity. While previous studies have identified bacterial enzymes associated with SA catabolism, no reported methods permit the selective labeling, tracking, and quantitation of SA-presenting microbes within complex multi-microbial systems. We combined metabolic labeling, click chemistry, 16S rRNA gene, and whole-genome sequencing to track and identify SA-presenting microbes from a cultured human fecal microbiome. We isolated a new strain of *Escherichia coli* that incorporates SA onto its own surface and encodes for the *nanT*, *neuA*, and *neuS* genes necessary for harvesting and presenting SA. Our method is applicable to the identification of SA-presenting bacteria from human, animal, and environmental microbiomes, as well as providing an entry point for the investigation of surface-expressed SA-associated structures.

## Introduction

Several methodologies and model systems have been critical to understanding gut microbiome taxonomic composition and the influence these compositions exert on host physiology and intestinal disease pathology^1–3^. Metagenomics and metabolomics as well as germ-free and gnotobiotic mouse models have helped establish how specific microbes and small-molecule metabolites modulate host responses to a variety of diseases^4–7^. These methods, in combination with the genetic manipulation of specific bacterial strains, have revealed key catabolizing enzymes and pathogen-associated molecular patterns (PAMPs) associated with microbiome-related metabolism, host immune activation, and bacterial infection^8–10^. Despite these methods and models, the ability to selectively label and track bacteria with specific catabolic functionalities in a highly complex and metabolically active microbiome remains limited.

*N*-Acetylneuraminic acid (Neu5Ac, termed sialic acid/SA here) is one of over thirty known sialic acid analogs. It is the predominant form of sialic acid in humans and is presented as the terminal residue on surface-exposed glycans, glycoproteins, and glycolipids^11^. SA is recognized by immunoinhibitory sialic acid-binding immunoglobulin-like lectins (Siglecs) to prevent autoimmunity^12^. Interestingly, select human pathogenic microbes have evolved to express this human SA epitope on their own cellular surface to evade host immune surveillance and clearance^8,13–15^. For example, group B streptococcus (GBS), a common cause of sepsis in human newborns, presents terminal α2,3-linked SA on its capsular polysaccharide (CPS) to bind Siglecs expressed by neutrophils, macrophages, and platelets and block immune activation^8,16,17^. Sequencing analysis revealed that GBS uses a tripartite transporter to translocate environmental SA onto its CPS to augment this immune evasion^8,18^. We posited that certain gut commensal organisms likely apply similar SA-mediated protective mechanisms to gain survival advantages. As such, the selective labeling and identification of these microbes from complex microbiome samples may reveal new SA-regulated host-microbe crosstalk mechanisms that could potentially be exploited for therapeutic development aimed at gut microbiome-related diseases.

Here, we report the application of an SA-based azide-containing probe *N*-acetyl-9-azido-9-deoxy-neuraminic acid (Neu5Ac9N_3_/Sia9N_3_), combined with flow cytometry and metagenomic and whole-genome sequencing, to selectively label, isolate, and identify SA-presenting bacteria from a complex cultured human fecal microbiome sample. Broadly, applications of metabolic probe labeling followed by click chemistry-tagging for visualization have been used to image and track pre-labeled bacterial components, including peptidoglycans and lipopolysaccharides^19–21^. However, there is limited use of SA-based probes to label bacterial glycans, especially in a highly complex microbial sample. With respect to eukaryotic labeling, “clickable” SA-based probes and its metabolic precursor (i.e., sialic acid-alkyne, sialic acid-azide, and mannosamine-alkyne) have been widely used for the study of sialoglycans in both in vitro and in vivo systems^22–26^. We sought to expand the application of SA-based metabolic probes to selectively label SA-presenting bacteria from a distal gut microbiome. Fecal microbiome samples collected from a healthy human volunteer were cultivated in the presence of the Sia9N_3_ metabolic probe^27^ and bacterial incorporation of Sia9N_3_ was examined by flow cytometry analysis following copper-free azide coupling with azadibenzocyclooctyne-conjugated biotin (ADIBO-BTN/DBCO-BTN) and streptavidin-Alexa 647 staining^28^. We identified a fecal microbiome sample from a healthy human donor containing bacteria that can readily incorporate Sia9N_3_. With fluorescence activated cell sorting (FACS) and 16S rRNA gene sequencing analyses, we found that the Sia9N_3_-incorporating bacteria belonged to the *Escherichia* genus. Isolation of the SA-incorporating bacteria via colony screening with whole-genome sequencing analysis suggested that the isolated bacteria is a new strain of *E. coli* that likely employs the NanT-NeuA-NeuS system to integrate environmental sialic acid to form polysialic acid on its capsular polysaccharide structure^29,30^.

## Results

### FACS-based screening helps identify microbiome constituents capable of Sia9N_3_ incorporation

**Figure 1:**
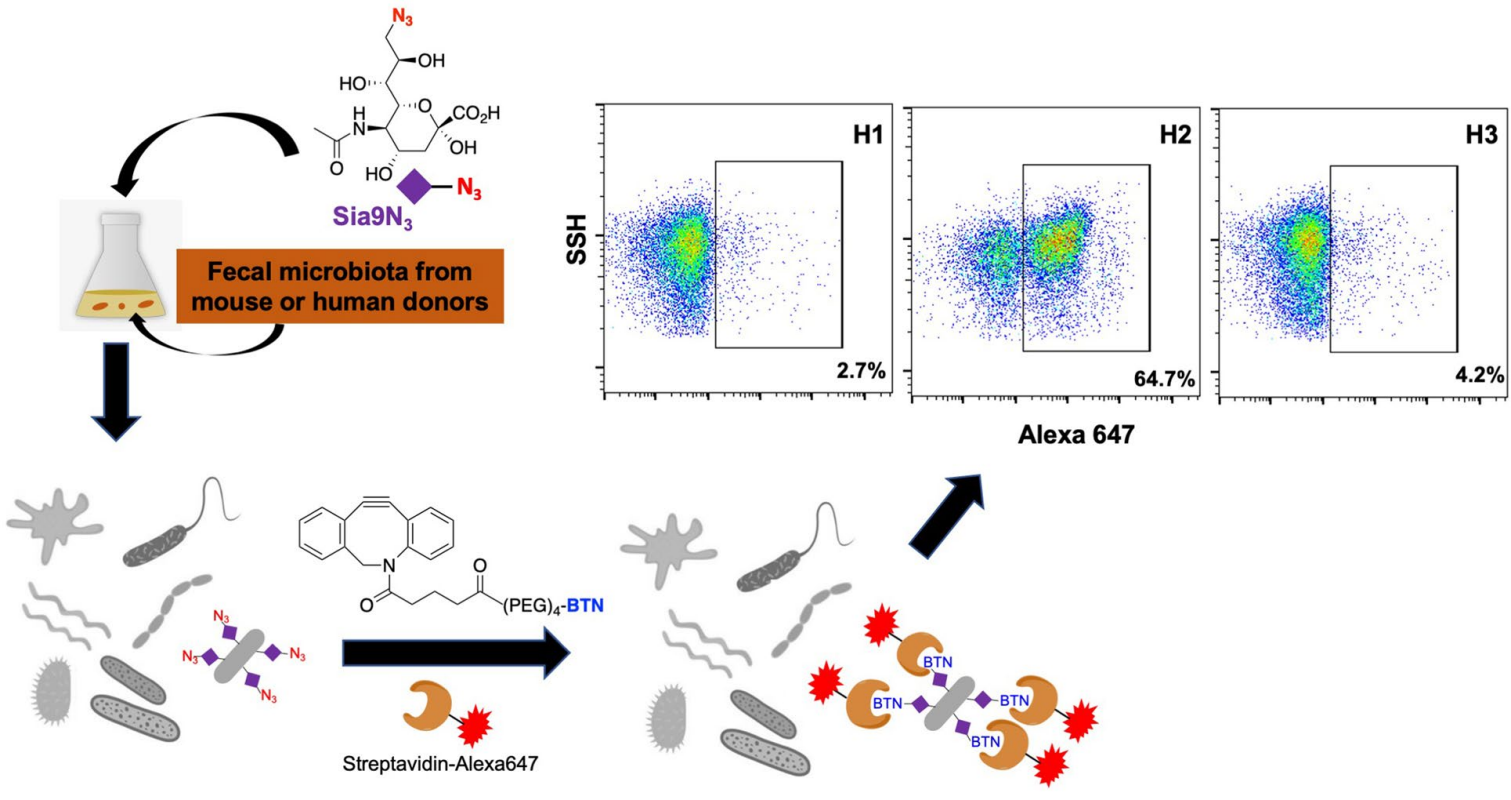
Metabolic labeling and isolation of SA-presenting microbes from a fecal microbiome sample. Fecal samples collected from a healthy human donor were cultured with the metabolic probe *N*-acetyl-9-azido-9-deoxy-neuraminic acid (Neu5Ac9N_3_/Sia9N_3_). The Sia9N_3_ incorporating bacterial cells were labeled with biotin through copper-free “click” reaction and were detected and isolated with flow cytometry.

**Figure 2:**
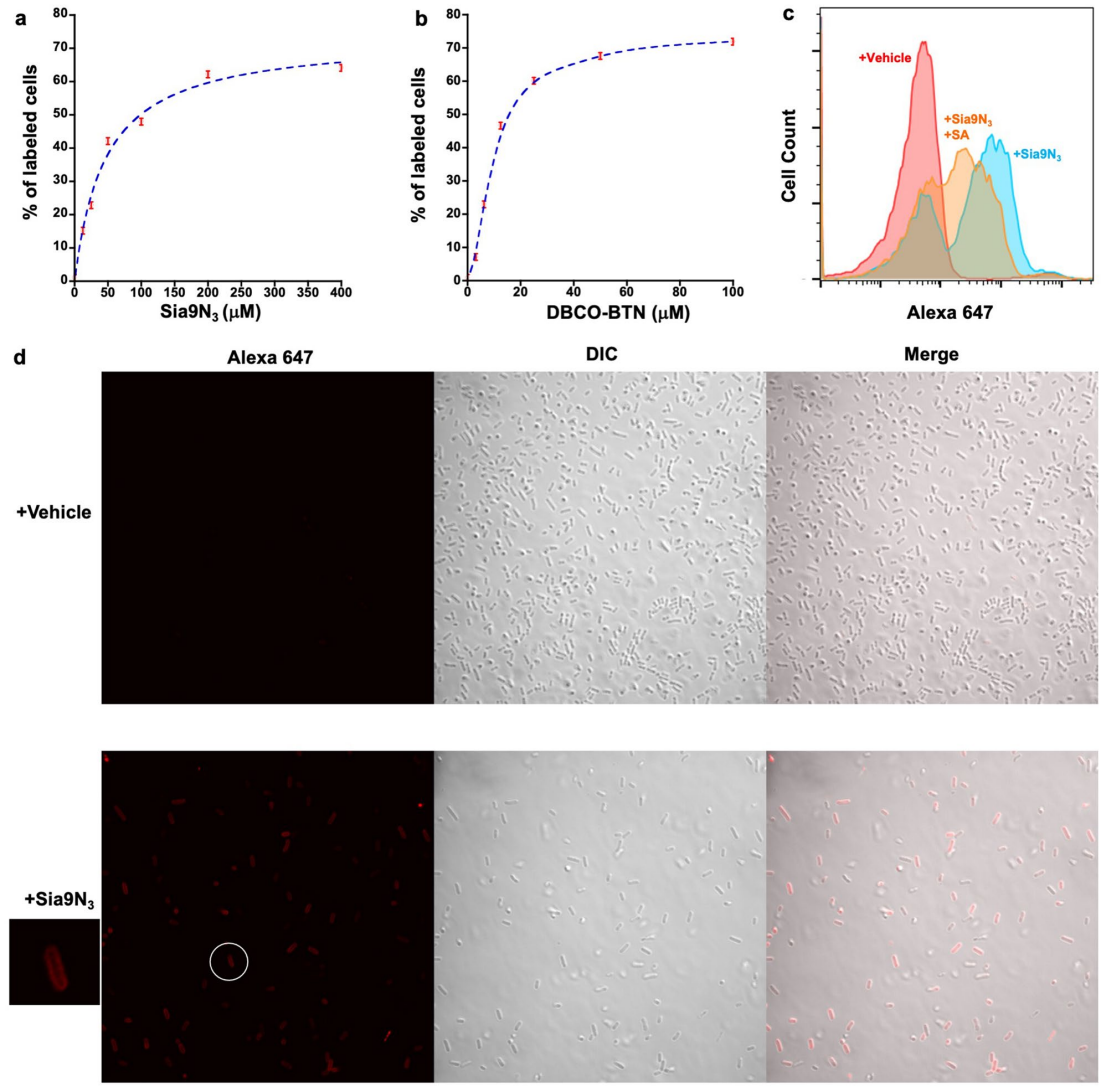
Characterization of Sia9N_3_ induced fluorescence staining with flow cytometry and confocal fluorescence microscopy. Labeling of SA-presenting microbes is Sia9N_3_ (**a**) and DBCO-BTN (**b**) dose-dependent. (**c**) Addition of excess natural sialic acid (SA) partially competed for fluorescence labeling ([Sia9N3]: 200 μM, [SA]: 1000 μM). (**d**) Confocal fluorescence microscopic imaging showed that Sia9N_3_ induced fluorescence labeling on the bacterial cell surface.

We surveyed fecal samples collected from one healthy human volunteer over the course of three months. Fecal microbes were cultured in a Gifu media^31^ supplemented with Sia9N_3_ at a physiologically relevant concentration (200 μM)^32^ under anaerobic atmosphere for 20 h. Microbes were centrifuged, extensively washed, subjected to DBCO-BTN conjugation with streptavidin-Alexa 647, and examined for fluorophore incorporation with flow cytometry analysis (Fig. 1). Among three microbiota samples tested (designated H1, H2, and H3), more than 60% of the bacterial cells from one of the cultured human fecal samples (e.g., H2) readily displayed fluorescence in response to Sia9N_3_ treatment (Fig. 1). Furthermore, a dose-dependent assay revealed that bacterial fluorescence labeling is dependent on concentrations of both Sia9N_3_ and DBCO-BTN. Use of 200 μM Sia9N_3_ in culture media with 50 μM DBCO-BTN for azide conjugation resulted in the maximum percentage of labeled bacterial cells (Fig. 2a,b). Further increases in the concentration of either labeling reagent only amplified the fluorescence intensity on the labeled bacterial cells without increasing background fluorescence or numbers of labeled cells, which suggested that Sia9N_3_ selectively labeled a subset of SA-catabolizing bacteria (Supplementary Fig. 1).

We next assessed if Neu5Ac would compete with Sia9N_3_ for surface expression by bacteria when supplemented into the 20 h cultures. Addition of excess Neu5Ac resulted in reduced labeling of bacteria by Sia9N_3_ (Fig. 2c; Supplementary Fig. 2a), which suggested that bacteria may use the same or partially overlapped transporters and enzymes for SA and Sia9N_3_ transportation and catabolism. Labeled bacteria were also subjected to fluorescence microscopic analysis to visualize where the bacteria are labeled (i.e., intracellular, periplasmic, cell wall, etc.). These experiments provided visual evidence that Sia9N_3_ or its azido-metabolites are prominently delivered to the bacterial cell surface (Fig. 2d). This finding is consistent with the observation that certain bacteria can present environmental SA onto its surface capsular polysaccharides and/or lipopolysaccharides^33^.

### Bacterial surface Sia9N_3_ is removed by microbial sialidases

**Figure 3:**
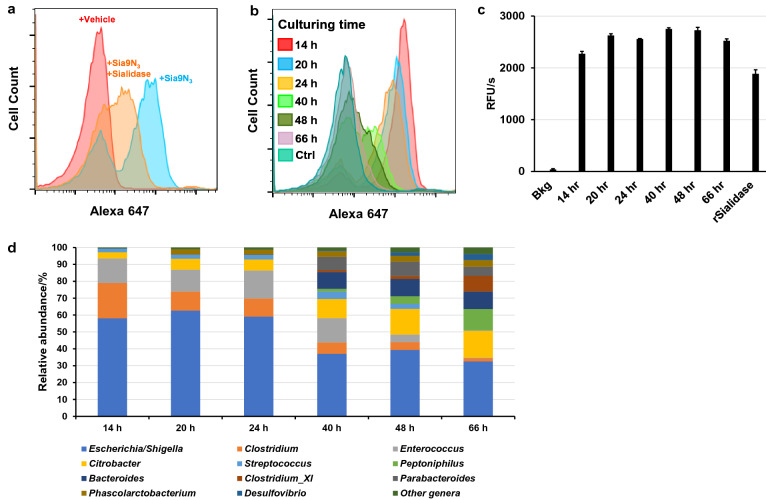
Bacterially incorporated surface Sia9N_3_ can be removed by microbial sialidases over time. (**a**) Treatment of labeled microbes before DBCO-BTN conjugation with a recombinant sialidase significantly reduces fluorescence labeling. The incorporated Sia9N_3_ is removed by microbial sialidases over time as fluorescence labeling was gradually removed during microbiota cultivation (**b**) and microbial sialidase activity was constantly detected in the culture media, 500 pM recombinant BT0455 was used as a positive control (**c**). (**d**) 16S rRNA gene sequencing revealed the change of taxonomic composition during H2 microbiome culture. “Bkg” stands for background, “rSialidase” stands for recombinant sialidase.

Many gut bacteria can express sialidases to release SA from sialoglycans, providing themselves or other bacteria with a source of carbon, nitrogen, and building blocks for cell wall biosynthesis^34,35^. To determine if the Sia9N_3_ probe is presented on bacteria to form sialoglycans, we treated the metabolically labeled H2 cultured sample with a purified recombinant sialidase (BT0455, NCBI gene ID: 1071627)^36^ from *Bacteroides thetaiotaomicron,* followed by sequential DBCO-BTN conjugation, streptavidin-Alexa 647 staining, and flow cytometry. Sialidase treatment dramatically reduced the number of fluorescently labeled bacterial cells and fluorescence intensity (Fig. 3a; Supplementary Fig. 2b). This result suggested that the majority of bacterial fluorescent labeling is due to Sia9N_3_ transported into certain bacterial cells and presented onto the cell surface to form sialoglycans. The inability to completely eliminate fluorescence labeling is likely due to substrate specificity of BT-0455 that includes α2,3-, α2,6-, and α2,8-linked sialic acid substrates^36^. Sia9N_3_ may be installed on glycans unrecognizable by BT-0455 and/or Sia9N_3_ is catabolized and surface expressed as a modified SA or other sugar.

Sialic acid can be released from sialoglycans by microbial sialidases as demonstrated (Fig. 3a), which can promote the growth of certain SA-utilizing pathogens in the gut and lead to inflammatory diseases^32^. To explore if Sia9N_3_ can also be released from the bacterially presented sialoglycans by other bacteria within the cultured sample, we tracked Sia9N_3_ incorporation over time during the plateau phase of bacterial growth. Briefly, the H2 fecal microbiome sample was grown with Sia9N_3_ for 14–66 h and the bacteria were quantitated for Sia9N_3_ incorporation by flow cytometry as described. Interestingly, detection of Sia9N_3_ labeling was maximally present at 14 h and gradually decreased until almost all labeling was eliminated by 40 h of incubation (Fig. 3b). Cultured media from each sample for every timepoint was assessed for sialidase activity, as measured by catalytic turnover of 4-methylumbelliferyl-*N*-acetylneuraminic acid (4-MUANA). Measurable sialidase activity was observed across all samples and suggests that bacterially secreted sialidases could remove Sia9N_3_ from bacterial sialoglycans over time (Fig. 3c). In parallel to the sialidase analysis, 16S rRNA gene sequencing showed that the compositional diversity of the cultured microbiota increased over time (Fig. 3d). At 14 h, the microbiome collection was dominated by *Escherichia/Shigella* (58% relative abundance), *Clostridium* (21%), and *Enterococcus* (15%). Common commensal organisms, including *Citrobacter, Peptoniphilus*, *Bacteroides*, and *Parabacteroides,* began to appear after 24 h of culture and their relative abundances peaked at 16%, 13%, 10%, and 5%, respectively, at 66 h of culture. The corresponding relative abundance of *Escherichia/Shigella*, *Clostridium*, and *Enterococcus* dropped to 33%, 2%, and 0%, respectively. The bloom of other commensal bacteria at later time points may suppress the detection of Sia9N_3_-presenting microbes, causing the elimination of fluorescently labeled populations. Of note, *Escherichia* and *Enterococcus* may be outcompeted at later time points during the anaerobic culturing as these bacteria are facultative anaerobes. It is also likely that complete elimination of Sia9N_3_ detection is due to sialidase activity. The sialidases responsible for the removal of Sia9N_3_ may not be present at early time points and are introduced by late-blooming commensal organisms, such as *Bacteroides*, that appear at later time points, as determined by 16S rRNA gene sequencing analysis. Importantly, this shift in taxonomic composition may reflect protective mechanisms by which commensal bacteria target virulence factors expressed on pathogenic/pathobiont bacteria by exposing surface antigens for host recognition. Alternatively, commensal bacteria could be modifying environmental conditions to reduce the growth of pathogenic/pathobiont bacteria. All together, these data demonstrated that Sia9N_3_ incorporation by bacteria in culture is dynamically regulated by other bacteria, nutrients, and bacterial sialidases.

### The isolated Sia9N_3_-presenting bacteria is a new strain of *Escherichia coli*

**Figure 4:**
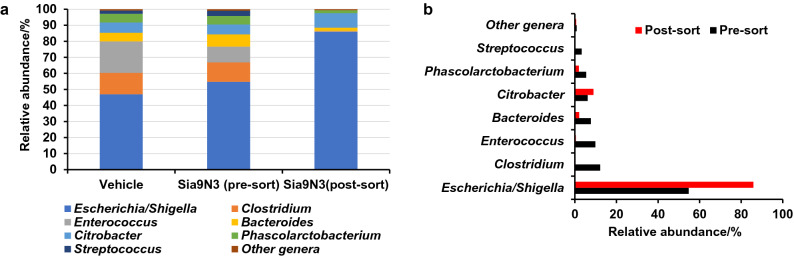
16S rRNA gene sequencing analyses. (**a**) H2 microbiome sample was cultured with or without 200 μM Sia9N_3_, which does not significantly alter taxonomic composition of 20-h H2 microbiome culture. Sia9N_3_ presenting bacteria from H2 microbiome culture were labeled with fluorescence and isolated with FACS. (**a**) and (**b**) 16S rRNA gene sequencing showed that the *Escherichia/Shigella* genus was significantly enriched after cell sorting whereas other species do not. This suggest that the fluorescently labeled cells are mostly *Escherichia/Shigella* bacteria.

We next separated the Sia9N_3_ incorporating bacteria from a 20-h culture of the H2 microbiome sample with FACS. The isolated fraction that yielded a high fluorescence signal due to Sia9N_3_ incorporation was subjected to 16S rRNA gene sequencing and revealed that the *Escherichia* genus was significantly enriched post sorting (Fig. 4). We next subjected the cultured and primary H1–H3 microbiome samples to 16S rRNA gene sequencing to determine if differences in the bacterial taxonomic composition accounted for the selective labeling of the H2 microbiome sample only. Of note, 20 h of culture significantly altered the composition of all samples, and therefore, made the Sia9N_3_-presenting bacteria detectable in H2 as the *Escherichia* genus was significantly enriched compared to the starting fecal sample (Supplementary Fig. 3). In addition, although only H2 contains Sia9N_3_-presenting bacteria, *Escherichia* dominated all three cultured microbiome samples as the most abundant genus. These results suggest that *Escherichia* presented in the H1–3 cultured microbiome samples may differ at the strain level as only the *Escherichia* in H2 has the catabolic genes to incorporate Sia9N_3_. An additional possibility is that the isolated *Escherichia* organism from H2 is present across all samples; however, the SA-presenting genes are not activated in the cultured H1 and H3 samples.

**Figure 5:**
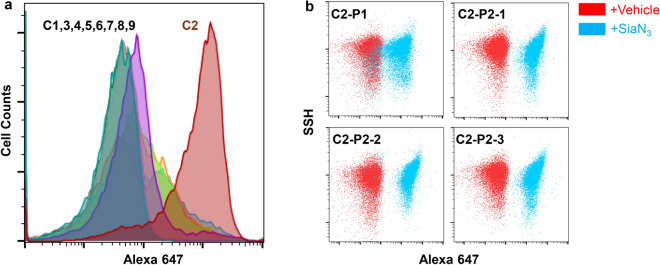
Isolation and passaging of the Sia9N_3_ incorporating bacterial strain from H2 microbiome. (**a**) Colony screening of H2 microbiome isolated one Sia9N_3_ incorporating bacterial strain. The raw H2 microbiota was diluted and plated on a Gifu-agar plate for overnight anaerobic culture, 10 colonies with different sizes and shapes were picked and cultured with the Gifu media supplemented with 200 μM Sia9N_3_ and subjected to fluorescence labeling study aforementioned. (**b**) The Sia9N_3_ incorporating feature is readily passaged to daughter generations. The cultured C2 media (C2-P1) was plated again and three colonies (C2-Passage 2, C2-P2-1,2,3) were picked for metabolic labeling study. Bacteria cultured from three colonies were almost 100% Sia9N_3_ incorporating cells, indicating that a single Sia9N_3_ incorporating bacterial strain is purified out from the microbiome complex.

To identify the bacterial genome of the Sia9N_3_-presenting H2 *Escherichia*, we cultured raw H2 microbiota on a Gifu agar plate overnight in anaerobic environment. Ten colonies were randomly picked and cultured with Gifu media supplemented with 200 μM Sia9N_3_ anaerobically for 20 h. The cultured colonies were subjected to click labeling and flow cytometry analysis. Out of 10 colonies screened, liquid culture of colony 2 yielded nearly 100% incorporation of Sia9N_3_ (Colony 2 Passage 1, C2-P1, Fig. 5a). Furthermore, 3 randomly picked colonies from a C2-P1 culture plate (C2 passage-2, C2-P2-1, -2, and -3) also showed 100% Sia9N_3_ incorporation, suggesting that the Sia9N_3_ catabolic genes were readily passaged to, and active in, the daughter generations (Fig. 5b). The ZH-C2 colony was subjected to shotgun whole-genome sequencing in an attempt to identify the *Escherichia* bacterial strain.

**Figure 6:**
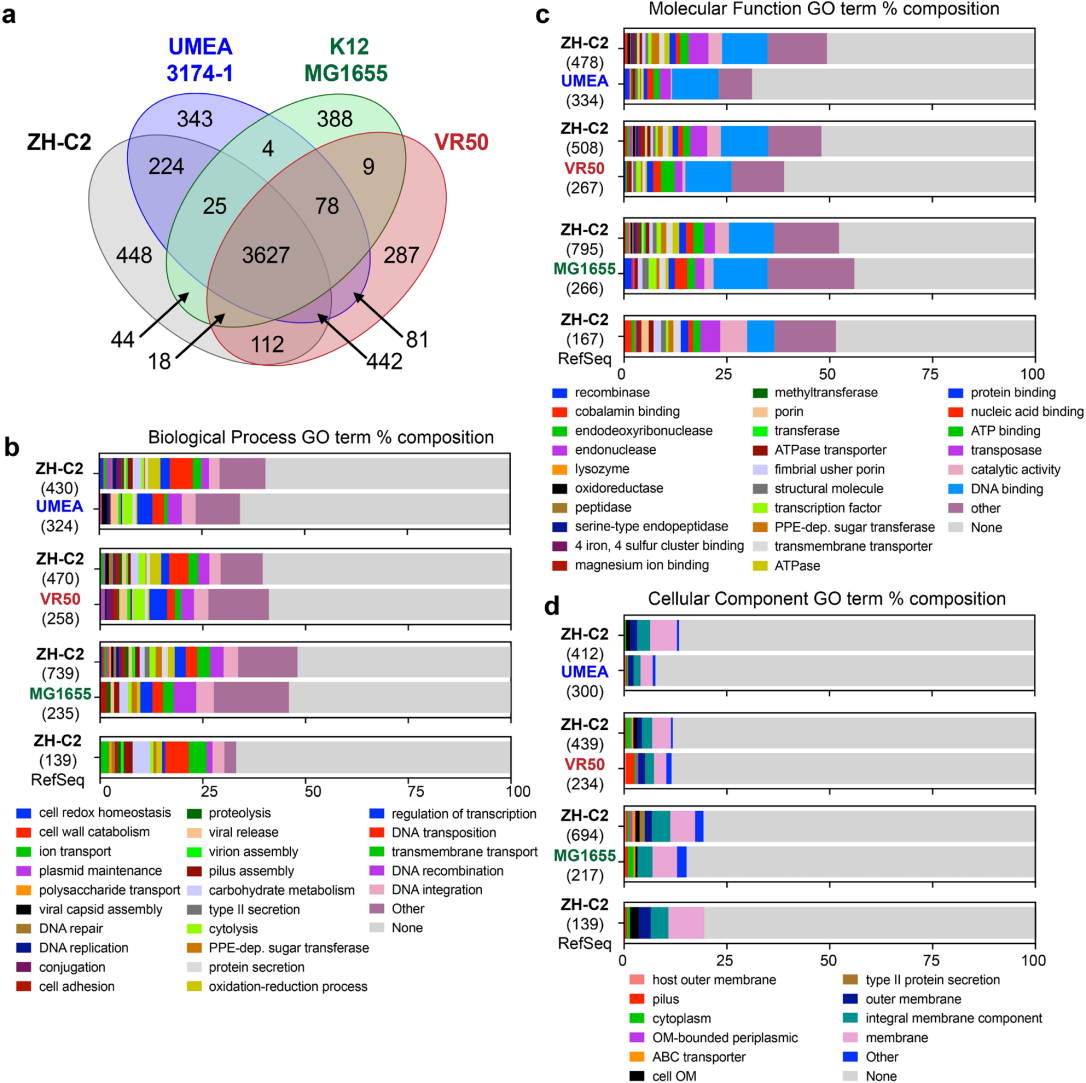
Comparison of encoded *E. coli* ZH-C2 genes to reference non-pathogenic and pathogenic *E. coli*. Venn diagram of CD-HIT analysis depicting conservation and differences of gene products between *E. coli* strains ZH-C2, K-12 MG1655, UMEA 3174-1, and VR50 (**a**). Protein sequences were clustered at CD-HIT sequence similarity cut-off of 90%. Biological process (**b**), molecular function (**c**), and cellular component (**d**) GO Terms of unique genes (less than 70% coverage and/pr less than 90% identity) found in ZH-2 compared to MG1655, UMEA 3174-1, VR50, and the RefSeq reference genome collection. The number of genes used for each GO term analysis is in parentheses. GO Terms listed as “other” include < 2 genes and are expanded in the Supplementary Table 1.

The genome of our newly isolated bacteria (ZH-C2) cannot be fully mapped to any previously sequenced *Escherichia* bacteria in the NCBI RefSeq database, but is most similar to pathogenic *E. coli* strains UMEA 3174-1 (accession PRJNA186306) and VR50 (accession PRJEA61445) with average nucleotide identities of 99.85% and 99.76%, respectively (Fig. 6a; Supplementary Table 1). Our *E. coli* ZH-C2 strain has a 5.23 Mb genome that is similar in size to UMEA 3174-1 and VR50 and is much larger than the 4.64 Mb genome of the prototypical nonpathogenic strain K-12 MG1655 (MG1655, accession PRJNA647163) (Supplementary Table 1). ZH-C2 shares over 90% identity in sequence to most MG1655 genes; however, ZH-C2 consists of 1111 missing or low-homology genes compared to MG1655, while MG1655 has 347 missing or low-homology genes relative to ZH-C2 (Fig. 6a; Supplementary Table 1). The majority of gene products have no identified function; however, GO term analyses demonstrate ZH-C2 encodes for an increased diversity and number of additional proteins relative to MG1655 involved in DNA repair and replication, polysaccharide transport, viron assembly, as well as type II secretion (Fig. 6b; Supplementary Table 1). With respect to molecular function, a diverse set of additional genes are encoded in ZH-C2, notably predicted cobalamin binding, ATPase, transferase, and porin activities (Fig. 6c; Supplementary Table 1) with a significantly larger collection of ZH-C2 proteins likely localized to the outer membrane compared to MG1655 (Fig. 6d; Supplementary Table 1). Conversely, the MG1655 genome consists of a number of additional genes associated with DNA-mediated recombination and integration, as well as cell adhesion, DNA binding and DNA recombinase activities (Fig. 6b–d; Supplementary Table 1).

MG1655 encodes for the sialic acid catabolizing gene cluster that includes *nanA*, *nanE*, *nanK*, the regulatory gene *nanR*, and sialic acid transporter gene *nanT*. However, in addition to these sialic acid-related genes, ZH-C2 also encodes for the polysialic acid biosynthesis (*kps*) cluster consisting of several genes, including: (1) *neuA*, the cytidine monophosphate (CMP)-sialic acid synthetase which produces the sialyltransferase donor CMP-sialic acid; and (2) *neuS*, the polysialyltransferase which synthesizes capsular polysialic acids from CMP-sialic acid^29^. MG1655 lacks *neuA*, *neuS*, as well as the other *kps* cluster genes, which highlights the inability of MG1655 to activate and present Sia9N_3_ on its cell surface (Supplementary Fig. 4). Moreover, the sugar-phosphate isomerase KpsF/GutQ (accession WP_001296394, ZH-C2_01729), known to be associated with polysialic acid deposition onto the capsule, is also identified in the ZH-C2 genome (with 100% identity to UMEA 3174-1 and VR50) but not in MG1655^37^. Altogether, the genomic analyses suggest that the cell-surface fluorescence labeling we observed is caused by deposition of polySia9N_3_ on the bacterial capsule.

Despite being isolated from a healthy human fecal sample, the genome of ZH-C2 strain is most similar to pathogenic strains UMEA 3174-1 and VR50, both of which were isolated from urinary tract infections (Supplementary Table 1). Established pathogenic genes, such as the type III secretion system are conserved across ZH-C2, UMEA 3174-1, and VR50^38^. Notwithstanding the high conservation, notable differences were also observed. ZH-C2 has 628 and 680 low homology/unique genes relative to UMEA 3174-1 and VR50, respectively. Conversely, UMEA and VR50 have 507 and 393 low homology/unique genes, respectively, to ZH-C2 as determined by sequences with < 90% identity and/or covering < 70% of the sequence length (Supplementary Table 1). Interestingly, there is an enrichment of predicted GO terms associated with DNA-mediated transposition, oxidation–reduction processes, pilus assembly, and carbohydrate metabolic activities in ZH-C2 relative to both UMEA 3174-1 and VR50, as well as a localization of proteins to the membrane (Fig. 6b–d; Supplementary Table 1). Those missing functions in ZH-C2 found in UMEA 3174-1 and VR50, include transcriptional regulation, viral release, nucleic acid binding, and recombinase activity (Fig. 6b–d; Supplementary Table 1).

With respect to all available non-redundant RefSeq reference genomes, ZH-C2 has 207 low homology or unique proteins with approximately 65 sequences consisting of < 100 amino acids (Fig. 6b–d; Supplementary Table 1). Almost all of these sequences have been identified in one or more of the other > 20 K *E. coli* strains that have been sequenced to date and are in the NCBI RefSeq/GenBank database. However, ZH-C2 encodes for ZH-C2_04397 and ZH-C2_04599 that are 1174 and 475 amino acids long, respectively, and have not been observed previously in any *E. coli* strain. ZH-C2_04397 is a multi-subunit protein consisting of an N-terminal autoinducer-2 kinase, an autotransporter barrel domain-containing lipoprotein, and a C-terminal HipA-like Ser/Thr kinase (Supplementary Fig. 5)^39,40^. While most *E. coli* encode for a linked autotransporter lipoprotein and C-terminal HipA-like Ser/Thr kinase, only one other *E. coli* has an N-terminal autoinducer-2 kinase connected to the autotransporter lipoprotein (LsrK in *E. coli* KTE98, accession EOV99460.1). However, this *E. coli* KTE98 gene lacks the C-terminal HipA-like Ser/Thr kinase. Most *E. coli* do encode for an autoinducer-2 kinase as a single gene. ZH-C2_04599 also is a multidomain gene consisting of an N-terminal transposase and a C-terminal EamA transporter (Supplementary Fig. 6)^41^. Again, both domains are well-represented across *E. coli* as single genes. The biological importance of these multi-functional connected domains will be subject to future investigations.

## Discussion

Azide-modified glucosamine, mannosamine, fucose, and 3-deoxy-d-manno-oct-2-ulosonic acid (Kdo) have been used to metabolically label bacterial cells in vitro and in vivo^19,20,42^. Very recently, Weiss et al. reported the application of functionalized monosaccharide derivatives including *N*-azidoacetylglucosamine, *N*-azidoacetylmannosamine, *N*-azidoacetylsialic acid in metabolically labeling of bacterial cells from murine intestinal microbiome^43^. Here, we focus on the application of azide-modified *N*-acetylneuraminic acid to selectively label and track Neu5Ac-presenting bacteria from a complex human fecal microbiome sample for the first time. Using flow cytometry and metagenomic sequencing, we identified a new strain of *E. coli* that can present environmental Neu5Ac on its surface via the NanT-NeuA-NeuS pathway. Our results also suggest a dynamic process by which select distal gut bacteria can exploit environmental sialic acid for self-decoration that, in turn, is likely subject to removal by sialidase activity provided by other microbiome constituents (e.g.,* B. thetaiotaomicron*)*.*

The Sia9N_3_-presenting activity of our new *E. coli* ZH-C2 strain is only detected in sample H2. Additional studies will investigate if this new *E. coli* is missing from the H1 and H3 cultures or present with a silenced NanT-NeuA-NeuS pathway during the ex vivo culture. Previous studies suggest both possibilities, as the gut microbiome is regulated by diet and crosstalk amongst bacteria as well as the host and suggests a dynamic composition of different bacterial strains over time^44,45^. Conversely, certain bacteria, including *Bacteroides fragilis*, are able to switch capsular polysaccharide structures enabling the colonization specific intestinal niches^46^. The gradual decrease in *E. coli* Sia9N_3_ surface expression over time will also be further interrogated. We anticipate that the loss of Sia9N_3_ is caused by other commensal organisms that gain prominence over time in the ex vivo culture and remove the *E. coli* poly-SA as a biologically relevant mechanism for host recognition and clearance. Additionally, surface-expressed poly-SA may be harvested by the parental *E. coli* for growth, as environmental conditions and nutrient availability shift over time.

Distal gut bacteria can degrade and ferment complex carbohydrates to provide an energy source and/or immunomodulatory molecules for themselves, the host, and/or other gut bacteria^1,32,47,48^. In return, host cells can produce glycans which can provide nutritional advantages for certain microbes^49^. Such mutualistic relationships make the tracking and study of carbohydrate catabolism by individual bacteria in complex microbiomes complicated and challenging. Consistent with previous studies, we found that cultured microbiome samples do not fully represent the taxonomic diversity of the original source due to growth advantages of certain bacteria under anaerobic systems with limited nutritional sources from the selected media^31,50^. Notwithstanding, our approach took advantage of the bias induced by bacterial culture and enriched for a new SA-presenting strain of *Escherichia coli* isolated from a human gut microbiome. We posit that SA-presenting bacteria can be identified from different microbial sources, including respiratory, urinary, and vaginal microbiomes in both health and disease (i.e., detection of pathogens in blood and stool clinical samples). Additionally, the scavenging of environmental sialic acid is associated with pathogenic bacterial virulence^51^, and proteins involved in the process may represent new targets for the discovery of antibacterial small molecules. Further, application of different probes, media, and culture conditions (i.e., pH, anaerobic/aerobic growth, time) may assist us in the identification of other bacterial species with catabolic functions of interest from clinically relevant microbiota samples.

Our integrated assay system detects sialic acid-presenting bacteria in real-time from complex microbiome samples. We envision that this labeling and tracking strategy can assist in identifying new SA-presenting bacteria and developing diagnostic tools and drug discovery models. Furthermore, this workflow may be used to investigate the installation of other environmental glycans by microbiota.

## Methods

### Synthesis, purification, and characterization of Neu5Ac9N_3_

ACS reagent grade organic solvents were purchased from Sigma Aldrich and were used directly if not specified. *N*-Acetyl-9-azido-9-deoxy-neuraminic acid (Neu5Ac9N_3_/Sia9N_3_) was synthesized, purified, and characterized as previously reported^27^. Briefly, Neu5Ac (3 g, 9.6 mmol) was mixed with Dowex 50Wx8-200-400 (H) (2 g, Alfa Aesar, L13922) in 50 mL methanol, followed by overnight stirring at room temperature. The resin was removed by filtration over Celite and the filtrate was concentrated under reduced pressure to 15 mL. 15 mL of ice-cold diethyl ether was layered above the concentrated methanolic solution, and the mixture was chilled at 4 °C overnight to yield a pale yellow crystalline solid. Supernatant was carefully decanted, and the pellet was carefully rinsed with ice cold diethyl ether twice, then air dried to give 2.2 g Neu5AcOMe as a white crystalline powder. Neu5AcOMe (2.2 g, 6.8 mmol) was dissolved in 20 mL of anhydrous pyridine, cooled in an ice bath, then treated with *p*-toluenesulfonyl chloride (2.35 g, 12.3 mmol; freshly purified by recrystallization from chloroform and petroleum ether). The mixture was stirred over an ice bath for 30 min, followed by overnight stirring at room temperature. Pyridine was removed by rotary evaporation to form syrup-like crude, which was purified with flash chromatography (EtoAc:MeOH = 20:1 ~ 20:5), dried, to yield 0.6 g Tosyl-Neu5AcOMe as white solid. Tosyl-Neu5AcOMe (0.6 g, 1.26 mmol) and NaN_3_ (0.32 g) were suspended in 10 mL of acetone:water (3:1) solution followed by overnight stirring at 70 °C. The crude was dried, acidified with Dowex 50Wx8-200-400 (H), and purified with flash chromatography (60:40 ~ 25:75 DCM:MeOH), dried to yield 0.31 g pure final product Neu5Ac9N_3_/Sia9N_3_.

### Sample collection

Human fecal sample collection methods were carried out in accordance with relevant guidelines and regulations. Experimental protocols were approved by the Institutional Review Board (IRB) at Scripps Research and Scripps Health; protocol #: IRB-14-635 and informed consent was obtained. Three human fecal samples (H1, H2, and H3) were collected from one healthy donor longitudinally at days 1, 18, and 80. Immediately after collection, fecal samples were transported in an anaerobic bag on dry ice into an anaerobic chamber (Coy Laboratory Products) filled with 3% hydrogen and 97% N_2_. Fecal samples were then re-suspended in filtered phosphate buffer saline (PBS) buffer, pH 7.4. Large non-bacterial particles were removed, and the cloudy supernatant was carefully aspirated out. Bacterial cells were then centrifuged down at 16,000×*g* for 5 min and washed with PBS 3 times. Fecal bacteria were cultured in the Gifu media in anaerobic atmosphere at 37 °C with an initial optical density at 600 nm (OD_600_) of 0.005. The rest of bacterial cells were re-suspended in 20% glycerol/PBS solution at an OD_600_ of 10, flash frozen, and stored at − 80 °C for future use.

### Sequential fluorescence labeling of bacterial cells and flow cytometry

Fecal microbes cultured with or without Sia9N_3_ were collected by centrifugation at 16,000×*g* and washed with filtered PBS for 3 times. Bacterial cells were then re-suspended to an OD_600_ of 1.0, treated with DBCO-Biotin for 2 h at room temperature in anaerobic atmosphere. The bacterial cells were washed with PBS for 3 times with centrifugation at 16,000×*g* for 5 min, followed by streptavidin Alexa 647 staining at 1:200 dilution for 1 h with OD_600_ at 1.0. Stained bacterial cells were washed with PBS for 3 times, followed by analyzing on a NovoCyte 3000 with NovoSampler Pro model. Singlet cells were selected by plotting SSC-H and FSC-H versus SSC-A and FSC-A, respectively, followed by gated on Alexa 647 channel. 15,000 total events were collected for data analysis with the percentage of singlets at ~ 90%.

### Fluorescence microscopy

Cultured H2 bacterial cells were thoroughly washed and subjected to click labeling with the biotin tag. Streptavidin Alexa 647 was then used to stain the cells. Fluorescently labeled bacterial cells were spotted on a glass slip, airdried for 15 min, and covered with a cover slip with ~ 1 mm thickness. Confocal microscopy was performed on a Zeiss LSM 880 Airyscan confocal laser scanning microscope. Imaging analyses were processed with FlowJo_v10.

### Expression and purification of sialidase BT0455

The gene fragment encodes for the *Bacteroides thetaiotaomicron* sialidase BT0455 (Uniprot ID: Q8AAK9) was ordered from Integrated DNA Technologies (IDT, with the N-terminal signal peptide removed. The BT0455 encoding sequence was cloned into a customized pT7HMT vector^52^, followed by expression in BL21(DE3) cells as an N-terminal His_6_ tag fusion. BT0455 was purified from bacterial cell lysate with a nickel–nitrilotriacetic acid (Ni–NTA) affinity column, followed by size-exclusion chromatography. Finally, BT0455 was aliquoted and stored at − 80 °C in a storage buffer containing 20 mM Tris–HCl and 100 mM NaCl with pH at 8.0.

### Sialidase activity assay

14–66 h after fecal microbiota culture, the bacterial cells were pelleted, and the supernatants (cultured media) were assayed for sialidase activity. Specifically, a 200 μL PBS mixture containing 500 pM purified BT0455 sialidase or cultured media and 100 μM turn-on fluorescent substrate 4-methylumbelliferyl-α-d-*N*-acetylneuraminic acid (4-MUNANA) was pre-incubate for 60 s, followed by fluorescence measurement on a microplate reader (excitation 365 nm, emission 450 nm). The fluorescence intensities were measured every 60 s over the linear range of enzymatic reaction. The presented data is representative of three individual activity assays. In each assay, fluorescence intensity at different time points were measured in duplicate.

### FACS

Fluorescence activated cell sorting (FACS) of fluorescently labeled bacteria from H2 microbiota culture was performed on a Sony MA900 multi-application cell sorter. Singlets cells were selected by plotting SSC-H and FSC-H versus SSC-A and FSC-A, respectively. Approximately 1 × 10^7^ fluorescently labeled bacterial cells were sorted and collected for 16S rDNA sequencing analyses.

### DNA extraction and sequencing analysis

Pre-sorted or post-sorted bacteria were subjected to DNA extraction with the ENZA bacterial DNA extraction kit (Omega biotek, D3350) based on manufacture’s protocol. 5–22 ng DNA was used with the NEXTFLEX 16S V4 Amplicon-Seq Kit 2.0 library prep kit (PerkinElmer) following manufacturer’s recommended protocol with between 11 and 20 PCR cycles depending on sample input. PCR amplicons were all pooled and gel purified to select the target amplicon region. Libraries were loaded onto a flowcell and sequenced on an Illumina MiSeq sequencer to generate 2 × 300-bp paired-end reads. We collected 0.2 and 3 million reads per sample for 16S rRNA and whole genome sequencing analyses, respectively. 16S rRNA gene sequencing data was processed with Illumina Basespace 16S metagenomic sequencing application for microbiome taxonomic annotation. The taxonomic classification step used ClassifyReads, a high-performance implementation of the Ribosomal Database Project (RDP) Classifier developed by Wang et al*.*^53^.

### Colony screening

Human microbiota H2 was diluted with PBS and plated on a Gifu-agar plate for overnight culture in an anaerobic atmosphere. 10 colonies were randomly picked and inoculated in 1 mL Gifu-Sia9N_3_ media for 20 h anaerobic culture at 37 °C. Then, bacterial cells were collected, washed, and tested for fluorescence labeling with flow cytometry as described. Bacteria cultured from C2 colony (C2 passage-1, C2-P1) showed nearly 100% fluorescence labeling. To test the homogeneity of C2 and C2-P1 bacteria, C2-P1 bacteria were cultured on a Gifu-agar plate and 3 colonies were randomly picked and inoculated in Gifu-Sia9N_3_ media followed by cultivation and fluorescence labeling experiments. As a result, 100% bacteria cultured from all three colonies (C2-P2-1 ~ 3) were fluorescently labeled.

### Shotgun whole-genome sequencing

Colony C2-P2-2 and C2-P2-3 were cultured in 6 mL Gifu media, respectively. Bacterial cells were then pelleted and washed, and bacterial DNA was extracted with ENZA bacterial DNA extraction kit (Omega Biotek, D3350) based on manufacture’s protocol. For genomic preps, DNA was sheared to ~ 500 bp size using a Covaris S2 Focused-ultrasonicator, after which 10 ng of sheared DNA was used to make sequencing libraries with the NEBNext Ultra II DNA Library Prep Kit following manufacturer’s recommended protocol with 9 cycles of PCR. Libraries were size selected using AmpureXP beads to 500–700 bp, loaded onto a flowcell, and sequenced on an Illumina MiSeq sequencer to generate 2 × 300-bp paired-end reads.

### Whole-genome assembly of ZH-C2

The reads for ZH-C2 were processed by Trimmomatic 0.36^54^ to remove the Illumina adapters and parts of the reads with low quality using the following filters: ILLUMINACLIP: < path-to-adapters > /TruSeq3-PE-2.fa:2:30:10 LEADING:30 SLIDINGWINDOW:10:20 MINLEN:100. The trimmed reads were then assembled using SPAdes 3.13.0^55^ with the following k values: 21, 33, 55, 77, 99, 127. Next, the blastn command of BLAST 2.7.1^56^ with -max_target seqs 1 was used to align the assembled ZH-C2 contigs against the NCBI RefSeq genome database (https://www.ncbi.nlm.nih.gov/genome). Two custom Perl scripts binned the voluminous BLAST output by species and strain. The average nucleotide identity (ANI) between ZH-C2 and the top-scoring strains was computed using blastn with -best_hit_overhang 0.1 -best_hit_score_edge 0.1 and another custom Perl script to determine the closest *E. coli* genomic strains: UMEA 3174-1 (closest overall), VR50 (second closest), and K-12 MG1655 (closest of three reference genomes). Some 11% of the ZH-C2 contigs did not align with *E. coli.* These were presumed to be from contaminants and were removed from the assembled genome along with another 5% of the contigs that were shorter than 200 bp to comply with guidelines for submission to GenBank.

### Identification of genes and their protein products

The proteins produced by UMEA 3174-1, VR50, and K-12 MG1655 are available from the RefSeq database. For ZH-C2, Prokka 1.14.6^57^ was used to identify the genes and associated proteins. Many were identified only as “hypothetical proteins”. To get further information on these as well as more consistent naming, a database of RefSeq bacterial proteins was generated using the commands given at https://dmnfarrell.github.io/bioinformatics/local-refseq-db. The blastp command of BLAST with -max_target_seqs 1 was then used to align the proteins from Prokka against the new database. This provided additional information on more than half of the hypothetical proteins examined; 19 proteins from Prokka were not found in RefSeq.

### Identification and removal of duplicate proteins

To check for the presence of duplicate proteins in a single genome, the proteins in each genome were aligned against themselves using blastp with -max_target_seqs 15. Most matches had less than 100% identity and 100% coverage, and these were ignored. The remaining perfect matches were mostly unique but also revealed 6 duplicate proteins in ZH-C2 from Prokka and 41 duplicate proteins in K-12 MG1655 from RefSeq. These duplicates were removed for subsequent analysis. No duplicates were found in the proteins for UMEA 3174-1 or VR50 from RefSeq.

### Comparison of proteins

The proteins in ZH-C2 were compared with those in each of the three other genomes of interest, and vice versa, using blastp. Even though -max_target_seqs 1 was specified, some proteins had more than one match with varying lengths. Thus, a second pass was made through the blastp output retaining only the longest match when there was more than one. Finally, the matches for each of the six pairwise comparisons were sorted into four groups corresponding to (1) perfect matches between the two genomes, (2) high-similarity matches with at least 90% amino acid identity covering at least 70% of the protein length, (3) low-similarity matches that did not meet the previous criteria, and (4) non-matches.

Venn diagrams were generated using output from CD-HIT (4.8.1) clustering of 4 combined proteomes (ZH-C2, MG1655, UMEA 3174-1, VR50)^58,59^. The following command line input was used to generate desired cluster files: “cd-hit -i inputfile.fasta -o outputfile -c %cutoff -g 1 -d 0” where “%cutoff” is the percent similarity cut-off used to define a cluster. In our analysis, we typically set percent similarity cut-offs at 65%, 75%, 85%, 95%, and 99%. CD-HIT output was organized into tables using the Python Pandas package and Venn diagrams were generated using venn/pyvenn packages.

Gene Ontology (GO) terms were generated using InterProScan (5.40-77.0)^60^. Briefly, protein sequences of interest were concatenated into a protein fasta file then submitted for InterProScan analysis using the following command line input: “./interproscsan.sh -i input_file.fasta -f tsv -dp -goterms”. Output tab separated files were organized using the Python Pandas package and bar plots were generated in Matplotlib. GO terms were mapped to their name spaces using the comprehensive “go.obo” listing available at geneontology.org^61^. GO terms were organized such that all protein sequences contributed at least 1 count to each of the 3 “Biological Process,” “Molecular Function,” and “Cellular Component” GO namespaces. If sequences could not be annotated, 1 count of “None” was contributed to each of the name spaces. For sequences annotated multiple times, each annotation contributed 1 count to the GO term’s respective namespace.

## Supplementary information

Supplementary information 1.

Supplementary information 2.

## Data Availability

The raw sequence reads and assembled genome are available from NCBI under BioProject PRJNA693345.
