# Supplementary material for: Identification of an N-acetylneuraminic acid-presenting bacteria isolated from a human microbiome
Source: Sci Rep. 2021 Feb 26;11:4763. doi: 10.1038/s41598-021-83875-w (PMC7910532; doi:10.1038/s41598-021-83875-w)
Supplement: Supplementary file 1 — Supplementary information 1. [file 41598_2021_83875_MOESM1_ESM.pdf]

Supplementary Information

Identification of an N-acetylneuraminic acid presenting-bacteria isolated from a human microbiome

Zhen Han[1], Peter S. Thuy-Boun[1], Wayne Pfeiffer[2], Vincent F. Vartabedian[3], Ali Torkamani[4,5], John R. Teijaro[3], Dennis W. Wolan[1,5]

[1]Department of Molecular Medicine, The Scripps Research Institute, La Jolla, CA 92037

[2]San Diego Supercomputer Center, University of California, San Diego, La Jolla CA 92093

[3]Department of Immunology and Microbiology, The Scripps Research Institute, La Jolla, CA 92037

[4]Scripps Research Translational Institute, The Scripps Research Institute, La Jolla, CA 92037

[5]Department of Integrative Structural and Computational Biology, The Scripps Research Institute, La Jolla, CA 92037

Supplementary Figures and Tables

Fig. 1. Dose-dependent labeling of H2 cultured microbiome sample.

Fig. 2. Sia9N_3 metabolic labeling of microbiota H2 competed with Neu5Ac and removed by a microbial sialidase BT-0455.

Fig. 3. 16S rDNA sequencing of primary and cultured microbiome samples.

Fig. 4. Sia9N_3 incorporation by *E. coli*-K12 (MG1655) and the newly isolated *E. coli* strain.

Fig. 5. Sequence alignment of ZH-C2_04397 to other *E. coli* proteins.

Fig. 6. Sequence alignment of ZH-C2_04599 to other *E. coli* proteins.

Supp. Table 1 7 tabs

Supplementary Table 1 legend: Additional bioinformatic data analyses of *E. coli* genome composition. Related to Figure 6. Tab 1: *E. coli* genome assembly statistics and comparisons to reference strains. Tab 2: List of low-homology and unique *E. coli* ZH-C2 genes relative to K-12 MG1655. Tab 3: Biological process, molecular function, and cellular component GO Terms associated with unique *E. coli* genomes. Tab 4: List of low-homology and unique *E. coli* ZH-C2 genes relative to UMEA 3174-1. Tab 5: List of low-homology and unique *E. coli* ZH-C2 genes relative to VR50. Tab 6: List of low-homology and unique *E. coli* ZH-C2 genes relative RefSeq collection. Low homology hit proteins are best match spans with <70% of query protein length and/or <90% positive identity rate. Tab 7: List of genes and protein products of ZH-C2.

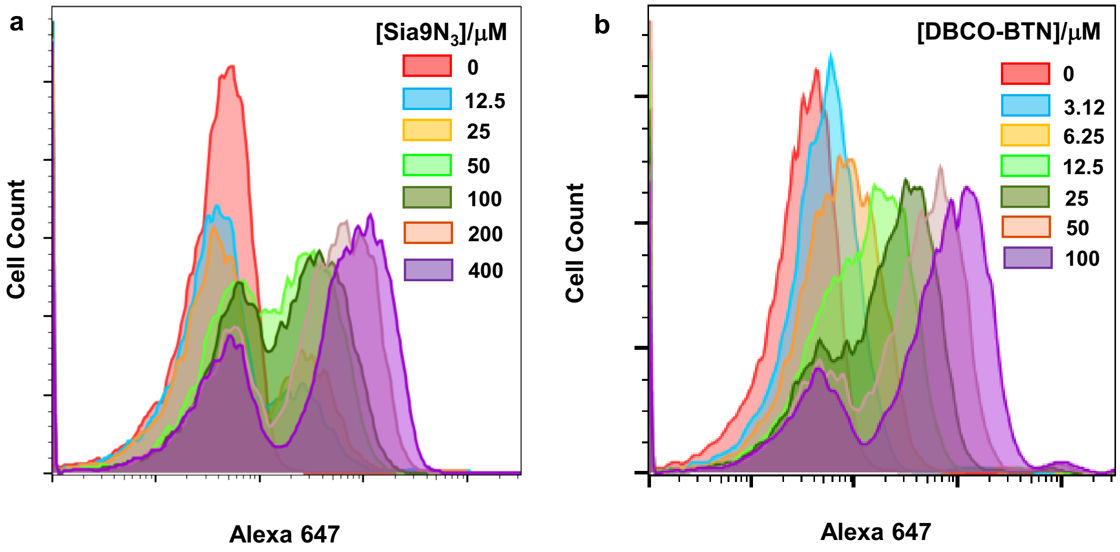

Fig. 1. Sia9N$_3$ (**a**) and DBCO-BTN (**b**) dose-dependent labeling of H2 cultured microbiome sample.

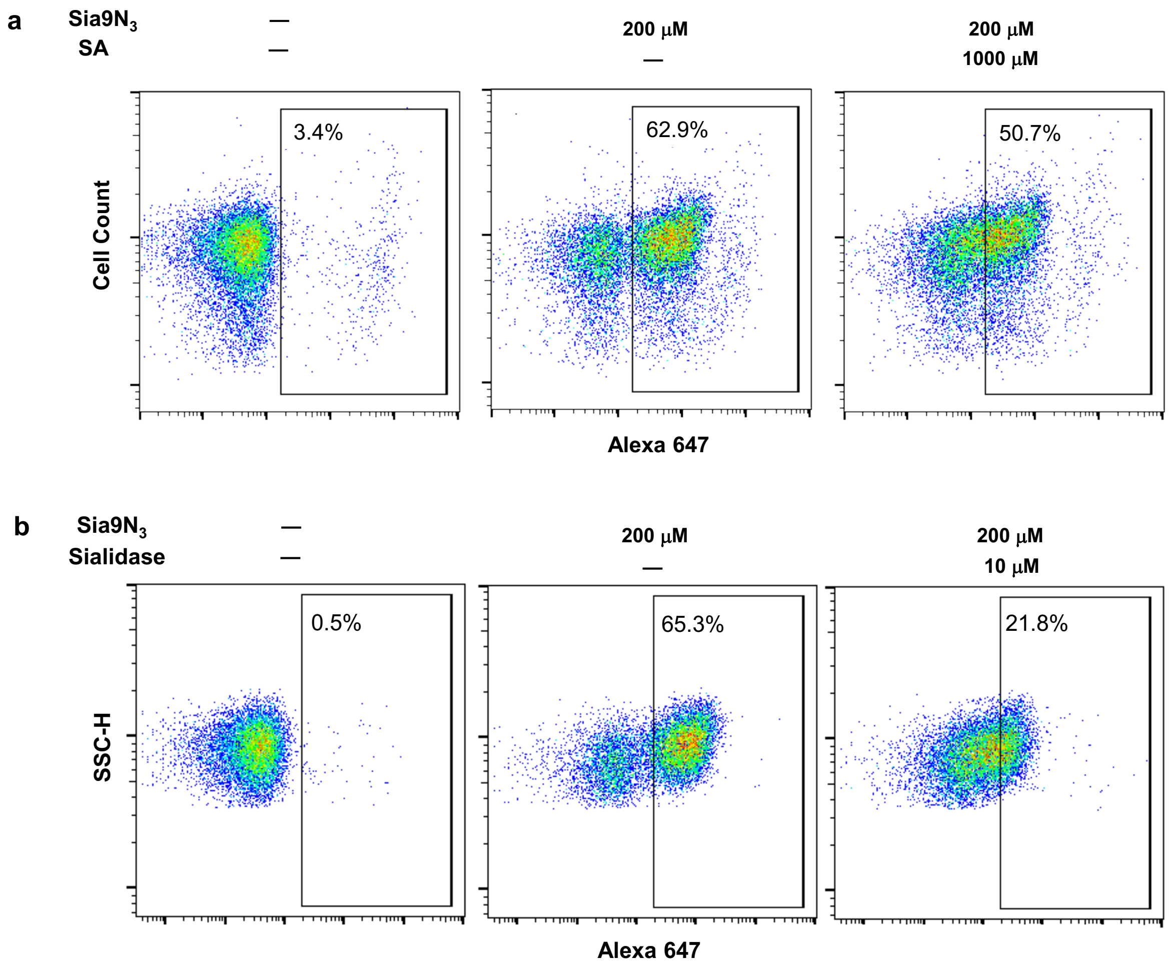

Fig. 2. a $Sia9N_3$ metabolic labeling of microbiome H2 competed with Neu5Ac. **b** $Sia9N_3$ metabolic labeling is partially removed by a microbial sialidase BT-0455.

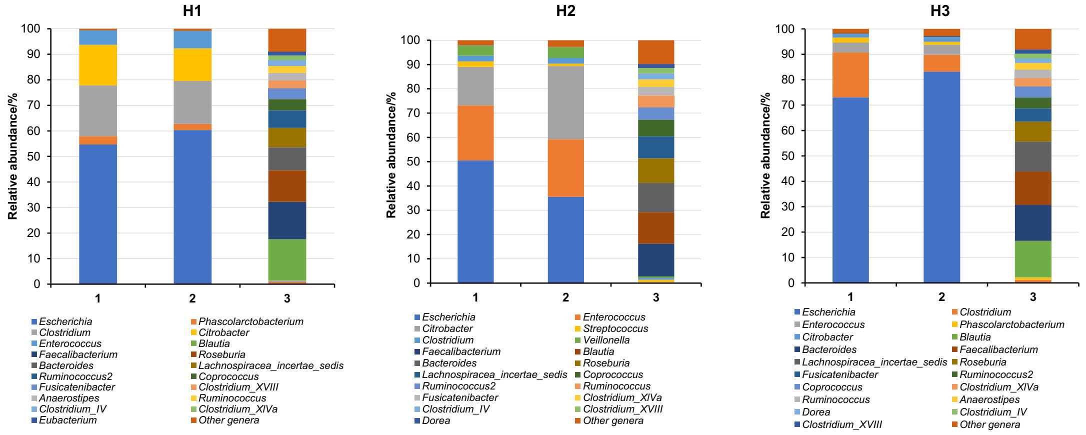

Fig. 3. 16S rRNA gene sequencing revealed taxonomic composition of cultured and primary microbiomes in the presence and absence of 200 μM Sia9N$_3$. Cultured human microbiome samples H1-H3 were all tested, column 1: microbiome cultured without Sia9N$_3$, column 2: microbiome cultured with Sia9N$_3$, 3: uncultured fecal microbiome samples.

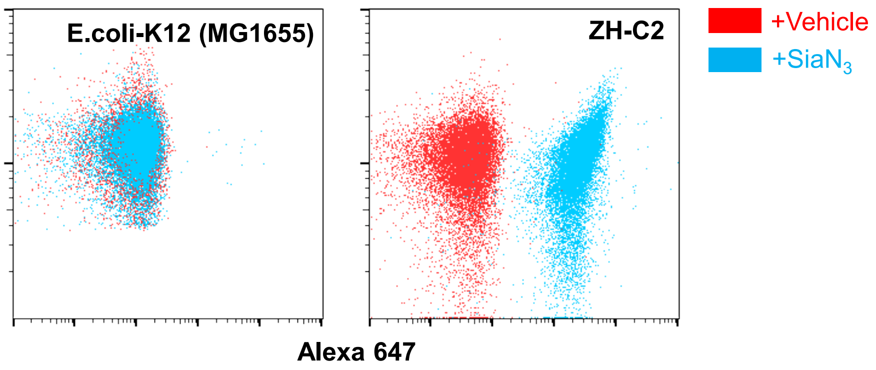

Fig. 4. Test of Sia9N3 incorporation by *E. coli*-K12 (MG1655) and the newly isolated *E. coli* strain ZH-C2.

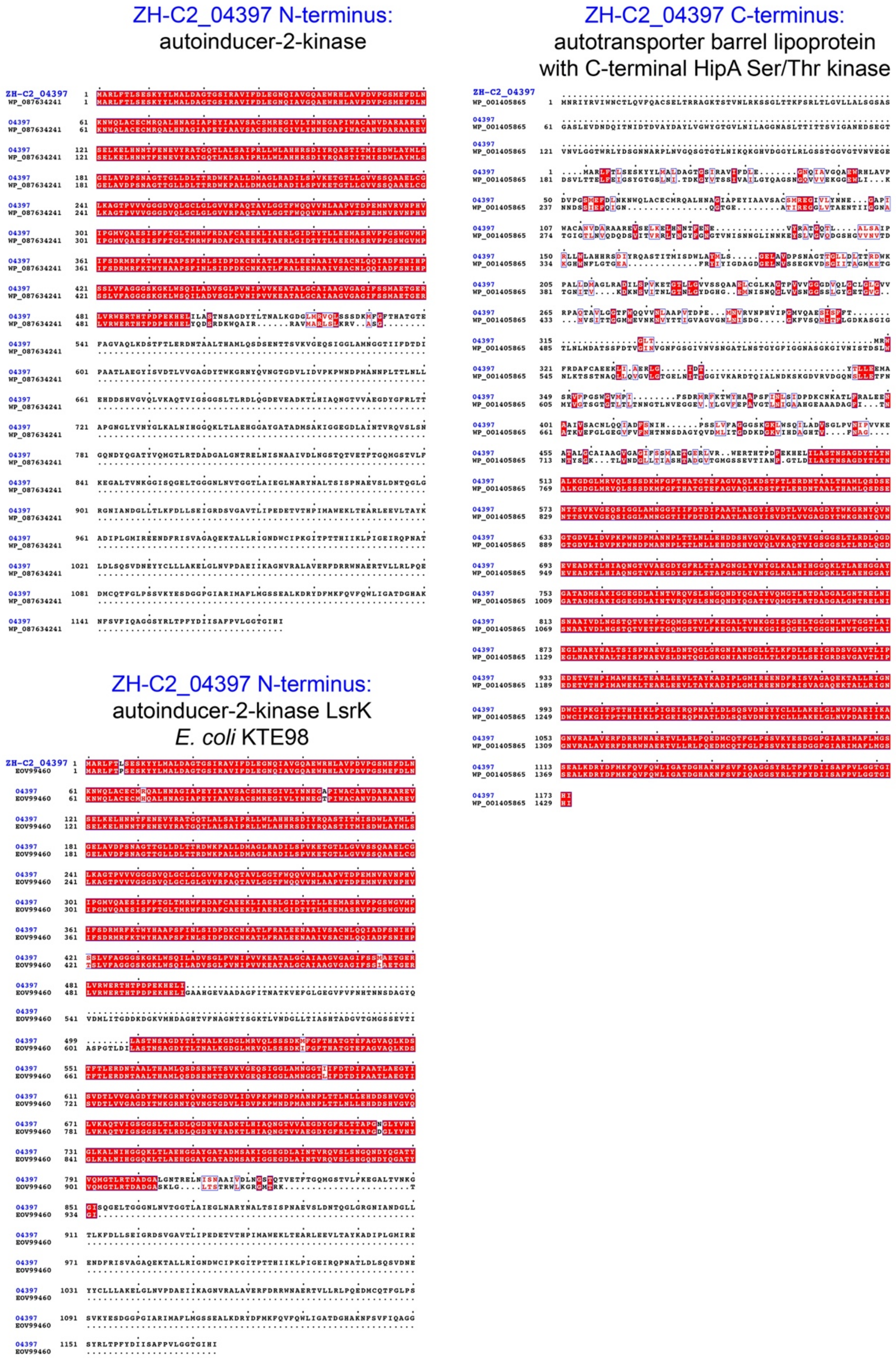

Fig. 5. Unique multi-domain ZH-C2_04397 with an N-terminal transposase, autotransporter barrel lipoprotein, and C-terminal HipA-like Ser/Thr kinase aligns to single-domain *E. coli* proteins.

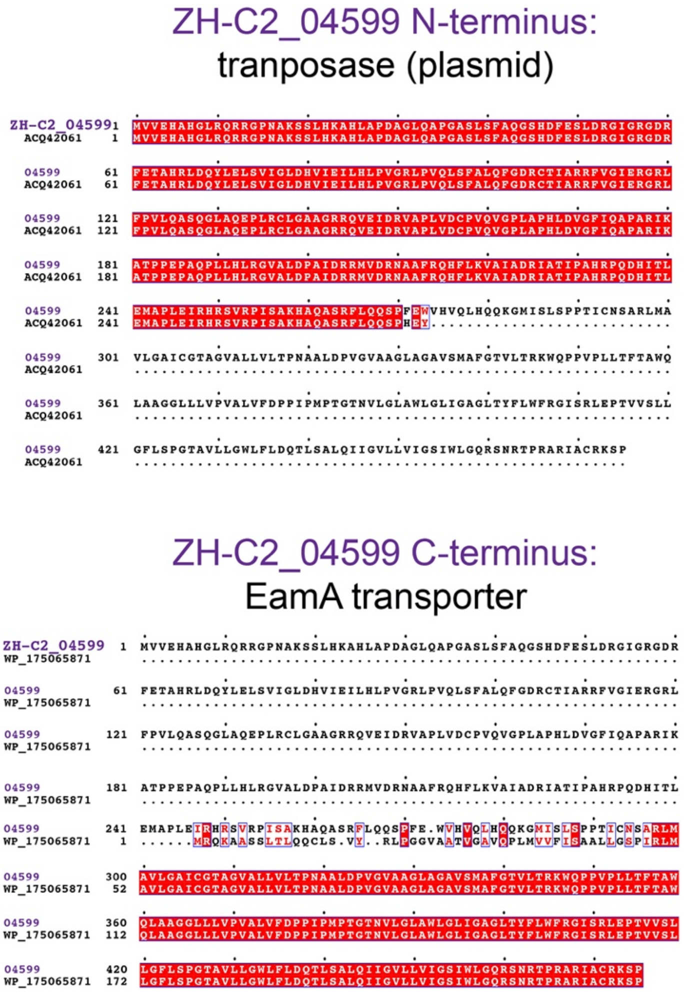

Fig. 6. Unique multi-domain ZH-C2_04599 with an N-terminal transposase and C-terminal EamA transporter aligns to single-domain *E. coli* proteins.
